# Enhancing Data Quality in Clinical Trials: Cross-Company Validation of the Open-Source Clinical Trial Anomaly Spotter (CTAS)

**DOI:** 10.1007/s43441-026-00950-y

**Published:** 2026-03-12

**Authors:** Pekka Tiikkainen, Frederik Collin, Björn Koneswarakantha

**Affiliations:** 1 Bayer Oy, Espoo, Finland; 2 Boehringer Ingelheim Pharma GmbH & Co, 88397 Biberach an der Riss, Germany; 3 Roche (Switzerland), Basel, Switzerland

**Keywords:** Quality assurance, RBQM, SDM, Time series, Anomaly, Clinical trial, Outlier

## Abstract

**Background:**

Current ICH guidelines, e.g. ICH E6 (R3), advocate a risk-based statistical review of clinical trial data to identify anomalies. The open-source R package, clinical trial anomaly spotter (CTAS) has been developed by Bayer and the Intercompany Quality Analytics (IMPALA) consortium, helps detect inconsistencies in subject time series data at both site and subject levels, facilitating timely intervention.

**Methods:**

CTAS analyzes time series of equal length. Each subject-level time series is summarized as six optional scalars: mean, standard deviation, range, relative unique value count, autocorrelation and local outlier factor. To detect site-level anomalies, sites can be scored using 3 different scoring methods. The performance of the CTAS algorithm was tested using simulations, artificially introducing site anomalies of various types and degrees into clinical trial data sets.

**Results:**

We found that CTAS can reliably detect site anomalies depending on the degree of the anomaly introduced. Less complex anomalies such as mean were easier to detect than complex outlier such as local outlier factor. The three scoring methods differed in their ability to detect anomalous sites with a small number of patients and their false positive rates.

**Conclusions:**

CTAS is a valuable tool for timely detection of outliers in clinical data, suitable for integration into risk-based strategies. Choosing the appropriate site anomaly scoring method is crucial for handling sites with fewer subjects effectively.

## Introduction

Most clinical trial data consist of repeated measurements taken of the same patients over the duration of the trial. The integrity of these data points is mostly challenged by systematic errors introduced at site level which can have root causes such as: misinterpretation of the protocol, miscalibration of laboratory tests or devices, insufficient staff training and many more. These errors will affect a larger portion of patients enrolled at the site and should be statistically detectable as site-level anomalies given that sufficient compliant data has been collected within the current trial data set. Classical risk mitigation strategies consist of a combination of monitoring activities such as source data verification (SDV) and source data review (SDR), programmed data management edit checks and medical data review.

Ensuring high data quality is a major responsibility of the clinical trial sponsor. The current ICH R6 (R3) guideline recommends a proportionate risk-based approach to survey and monitor the completeness, expected range and variability of the clinical trial data [1]. Typically, clinical data quality is measured against expectations that are derived from intrinsic knowledge of the data generation process [2]. To counter unexpected anomalies, unsupervised statistical data monitoring (SDM) methods can be employed. It has been shown that SDM can improve data quality over time [3]. SDM is usually integrated into a risk-based quality monitoring (RBQM) framework which also includes tracking of site key risk indicators (KRI) derived from operational metrics. Recently, an RBQM open source solution for KRI monitoring has been developed [4]. Currently we are not aware of any open source solution for SDM.

In this paper, we present an evaluation of the Clinical Trial Anomaly Spotter (CTAS)—an open-source R package for the identification of anomalous clinical trial time series. The package was developed originally by Bayer and later co-developed by the Intercompany Quality Analytics (IMPALA) consortium. IMPALA has currently 19 industry members and promotes the use and development of analytics for GxP quality assurance [5]. CTAS fills the central statistical data monitoring gap of the current open source RBQM landscape. It has foremost been developed for the detection of anomalies in numerical time series data at site and subject level. The strong focus of CTAS on time series data ensures that only time series of the same length are being compared, reducing false positive signals.

In this article, we demonstrate the efficiency in detecting anomalous sites using simulations based on data from 15 clinical trials donated by three of the IMPALA member companies.

## Methods

### Algorithm

#### Clinical Trial Time Series

In a clinical trial, patient data is typically collected as repeated measures over time, tracking pivotal health measurements of all patients during the course of a trial. As patients can enter the trial at different times and thus the individual length of each time series depends on the time that has passed since enrollment. The number of data points collected depends on how often a measurement was collected and may contain missing values when assessments have been skipped. The actual values measured may also depend on scheduled treatments and assessment as well as on disease progression. All these factors complicate active SDM of ongoing trials. CTAS has been developed to only compare time series composed of the same time points. It will actively comb through the data and find as many groups of timeseries of equal length that fulfill a set of parametrized minimum requirements. Detailed parameter descriptions can be found in the project’s public code repository (see Supplementary Information). Alternatively, time series can also be defined manually to include fixed sets of visits. CTAS expects measurements to be numerical, thus any categorical values need to be transformed to an appropriate numerical format. CTAS will then calculate a set of relevant aggregate features for each time series which can then be used to identify site and subject outliers.

#### Defining Time Series

**Fig. 1:**
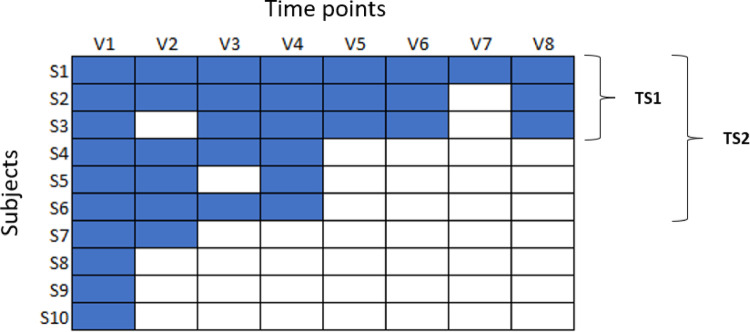
Simplified example of time series group definitions. Blue cells denote subjects with a measurement at a given time point. Two time series groups have been defined. TS1 includes subjects (S1–S3) which have finished the study TS2 focuses on the first four time points and includes also subjects S4-S6 in addition to those in the TS1. Remaining subjects have data for too few time points to be considered in either of the time series groups

In an ongoing study, some subjects might have only taken the first visits while some have already finished the study. For this reason, more than one group of time series with various lengths per parameter should be defined. One group would include almost all planned time points and compares subjects which have largely finished the trial. Another shorter group includes only the first few visits to also include subjects which have been recently enrolled. As illustrated in Fig. 1 individual subject data points may also be missing. These time series groups can be manually defined or CTAS will start to iteratively look for suitable groups starting with the longest possible group and decrease the length if the number of eligible subjects can be increased by at least 20 percent. Regardless of whether timeseries groups are defined manually or automatically timeseries groups of different length will be evaluated simultaneously by CTAS to balance length and the number of patients. Figure 1 illustrates two timeseries groups TS1 with 3 patients of length 8 and TS4 with 6 patients of length 4. A time series group should have a minimum group size which can be set via a CTAS parameter, we currently recommend a minimum group size of 25 subjects. Eligible subjects must also not exceed the maximum ratio of missing values, which is another CTAS parameter for which the recommendation is 30%. Traditionally, time series should have at least three data points, however some measurements will only be taken once possibly during screening. Therefore, CTAS also allows time series of length one, although not all subsequent time series features e.g. autocorrelation (see the following section) will be meaningful.

#### Timeseries Features

Each time series is characterized by a set of time series features, which summarize the measurements of a time series into a single metric. These features are central to the algorithm as they are used to flag anomalous sites (see below). In addition, the features can be used to identify individual subjects with peculiar time series. Available time series features are listed in Table 1.

**Table 1:** Time series features

Feature	Anomaly Example
Average value	Systematic bias from lab calibration issues, resulting in shifted measurements
Standard Deviation	Implausible fluctuations in liver enzymes that may indicate a safety or technical issue
Range	Magnitude errors caused by incorrect unit assignments (e.g., mg/dL vs. mmol/L)
Unique Value Ratio	Data duplication or rounding, identified by a lack of variety in continuous measurements
Autocorrelation	Artificial linearity caused by sites adding a constant value to previous measurements
Local Outlier Factor (LOF) [6]	Extreme patterns (LOF > 1) or suspicious uniformity (LOF ~ 1) where the latter would suggest fabricated "normal" values

All feature types except LOF (local outlier factor) [6] are context-free meaning that only measurements in the time series are needed to calculate them. LOF on the other hand is context dependent as its value depends also on the other time series in the study.

**Fig. 2:**
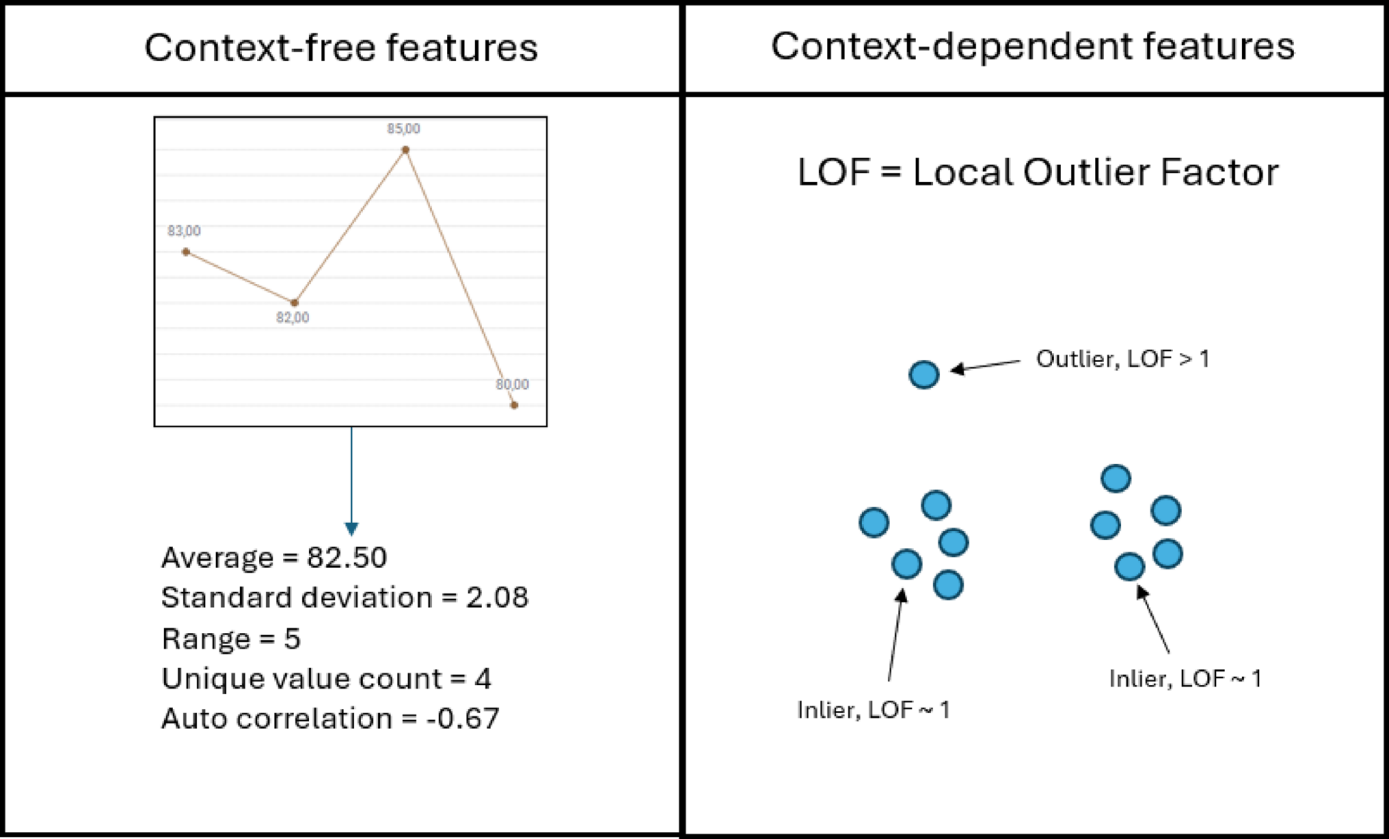
Example of a time series with four time points and the features calculated from it. The context-free features can be calculated by using the data in the time series itself. In contrast, Local Outlier Factor (LOF) are context-dependent, and its value depends on the time series of other subjects in the study

For an illustration of features for a time series, please see Fig. 2.

### Flagging Sites

Flagging sites with a systematic bias in their data collection is an important part of study monitoring. If identified early enough, the site can be offered further training if the site has had trouble in interpreting the study protocol. In the extreme case, if the bias is due to intentional misconduct, the site can be closed and excluded from analysis.

Sites are flagged based on the bias in their time series feature values versus other sites in the study. CTAS offers three options for flagging sites: Kolmogorov–Smirnov, mixed effects modelling and the simple average of the feature values. P-values derived from those methods are corrected for multiplicity using the Benjamini Yekutieli correction accounting for interdependency between samples as data is reused between control groups [7]. The negative logarithm of the corrected p-values provides the CTAS score which is provided for every timeseries feature and time series group. We used the maximum score from all predefined or autogenerated time series groups with a threshold of 1.3 (*p*-value: ~ 0.05) to flag site outliers.

#### Kolmogorov–Smirnov

The Kolmogorov–Smirnov (K-S) statistical test compares time series feature values of individual sites against the combined values from all other sites. This non-parametric test evaluates the null hypothesis that the feature values from a specific site and the feature values from all other sites are drawn from the same distribution [8].

The K-S test calculates the maximum difference between the empirical cumulative distribution functions (ECDFs) of the two samples, providing a D-statistic and a p-value for each comparison. The p-value indicates the significance of the difference between the distributions. A low p-value suggests that the feature values from the site are significantly different to those from other sites, potentially indicating an anomaly.

**Fig. 3:**
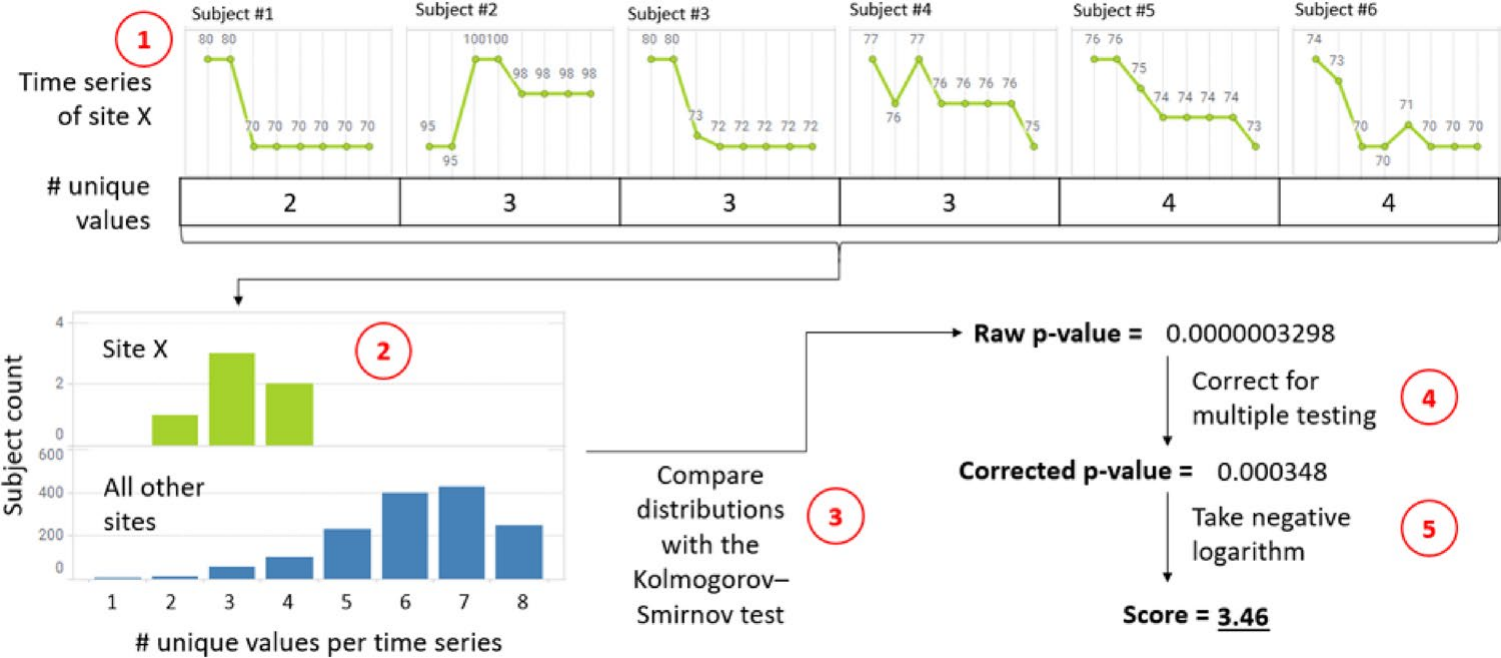
Example of site flagging. The timeseries has eight time points and the question is whether the site (six subjects) has reported fewer unique values per time series than other sites in the study. Part (1) has the individual subject time series and the unique value counts. It is clear from the histograms (2) that the site is biased when compared to other sites. To quantify the bias, the distributions are compared with the Kolmogorov–Smirnov test (3) which gives us a raw p-value. As we perform several tests per study, the *p*-value must be corrected (4). Finally, the negative logarithm of the corrected *p*-value is taken to come up with the final score for the site (5)

Figure 3 gives an example on how the method is used to identify a site with few unique values per time series.

#### Mixed Effects Modelling

This approach allows us to account for both fixed and random effects in the data, providing a robust framework for detecting anomalies.

We fit a mixed effects model to the data, where the hierarchical structure of the data is taken into account by including random effects for different levels (i.e. sites nested within countries and regions). After fitting the model, we simulate the random effects to obtain estimates for each entity (sites, countries, and regions). For each entity, we calculate the median and standard deviation of the random effects. Consequently, the median and standard deviation are used to calculate a p-value for each entity’s deviation from zero, providing a statistical measure of the significance of the anomaly.

#### Average Feature Value

As a simple baseline model for anomaly detection, we also scored sites based on the average feature values of the subjects at each site. Then outliers were defined as being outside of the interquartile range multiplied by 1.5 [9]. This straightforward approach involves calculating the mean feature value for each site and using these averages to identify potential anomalies. It is included to have a benchmark to see if the more complex site flagging methods are able to outperform it. Note that the method does not produce a p-value but a binary outcome on whether the site is an outlier or not.

The primary advantage of this method is its simplicity and ease of understanding. It provides a clear and straightforward way to identify sites with unusual feature values. However, this approach does not take into account the size of the site. A site with only one subject who has an extreme feature value may get flagged, even though this could be due to chance alone and may not indicate a systematic issue with the site.

### Identifying Anomalies in Subject-Level Time Series

**Fig. 4:**
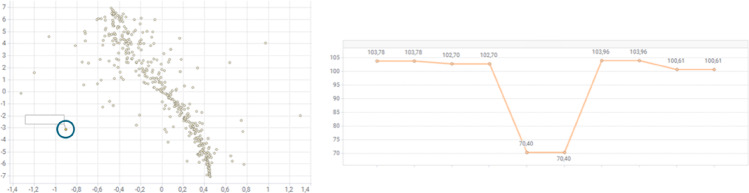
An anomalous weight profile identified based on its distance from other profiles on the similarity plot (left). The profile is given on the right with a sudden drop in weight followed by a return to the previous values. In this case, the reason is probably a data entry error at site

**Fig. 5:**
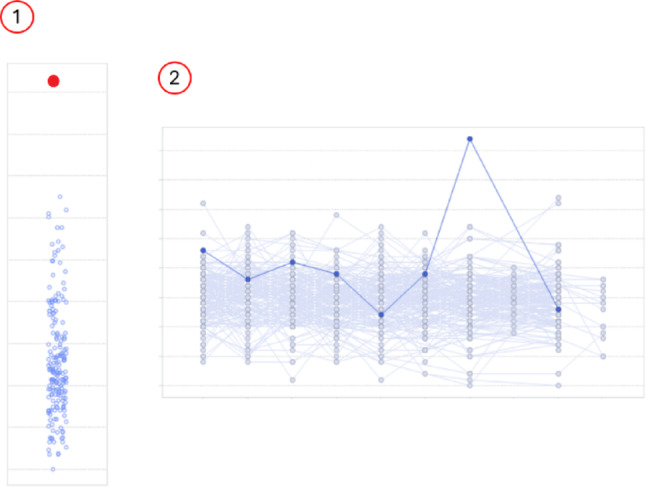
Identifying an individual time series outlier based on a time series feature. In this case the subject with most variable bilirubin profile (1) has been selected and highlighted with the other subjects (2). In this case, it is possible that the site has collected the data correctly, but this might be interesting for someone performing medical review to identify safety issues, for example

In addition to flagging sites, the results are valuable for identifying individual subjects with anomalous time series. Sites which exhibit no systematic bias might still contain individual anomalous subjects and time series. One way is to visually inspect similarity plots for subjects with few near neighbors. Figure 4 gives an example of a subject-level outlier with an anomalous weight profile. Another approach is to compare time series features and review subject time series with extreme values for one or more features. Please see Fig. 5 for an example. Note that outliers identified with the two approaches often correlate, e.g. subjects with unusually high standard deviations also tend to be outliers on the similarity plot.

### Validation

The primary goal of the evaluation is to demonstrate CTAS performance to detect outliers of varying degrees in a clinical trial data set using the different statistical methods offered by CTAS (mixed effect modelling, K-S, or average feature value) for flagging site-level outlier. Secondarily, we check whether CTAS is suitable for different types of clinical time series measurements: two laboratory (Alanine Aminotransferase, Creatinine) and two vital sign measurements (Systolic Blood Pressure, Weight). We created test data sets from clinical trial data sets from which we removed potentially preexisting site-specific signal as well as regional and country-specific effects by reassigning all patients to different sites.

Further we removed data points from unscheduled visits and data from screening failures. A total of 15 completed studies were selected from 3 different IMPALA members. These studies were divided into groups of three studies with each group having comparable numbers of patients and sites.

In order to simulate detectable anomalies, we randomly selected three sites to which we applied the following transformations:

- Average: Add the site mean multiplied by the anomaly degree to original values.
- Standard Deviation: Add site mean multiplied with the anomaly degree to each observation and randomly apply a negative or positive fore-sign.
- Range: Add one outlier data point per patient. The extremity of the outlier is based on the anomaly degree.
- Unique Value Ratio: Replace a ratio of observations with the first observed value per patient. The ratio would be determined by the anomaly degree.
- Autocorrelation: Add the preceding value multiplied by the anomaly degree to each value.
- Local outlier factor: Transform data for each patient by a randomly chosen non-normal distribution. The anomaly degree would determine the ratio of affected patients.

**Fig. 6:**
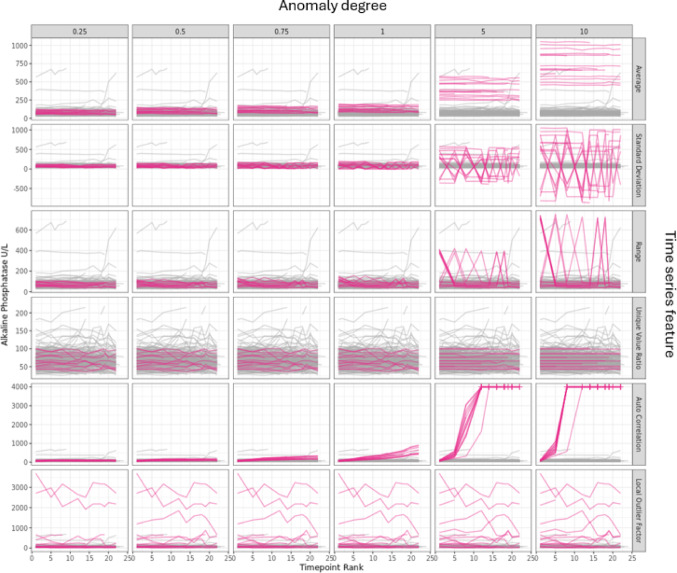
Examples of simulated site-level anomalies (for Alkaline Phosphatase) of varying degree across different anomaly types based on a simulated test data set. Timelines from regular sites are shown in grey and timelines from anomalous sites in red

The anomalies introduced are visualized in Fig. 6 based on a simulated Standard Data Tabulation Model (SDTM) test data set [10]. These anomalies pair with the time series features that are measured by CTAS and listed in Table 1 which also gives examples of how they might occur in a clinical trial setting. The simulated anomalies mimic these events to different degrees.

Subsequently we tried to detect the three anomalous sites using different site scoring methods among the compliant sites in the study data set. We set the minimum time series length to one and required at least 25 patients per time series group with a maximum ratio of missing values of 30% per subject. For each combination of a site, parameter and feature, we took the highest CTAS score, across all potential time series groups and employed a threshold of 1.3 (*p*-value: ~ 0.05). This was repeated a 100 times and true positive rate (TPR) and false positive rate (FPR) were calculated using the combined results.

## Results

**Fig. 7:**
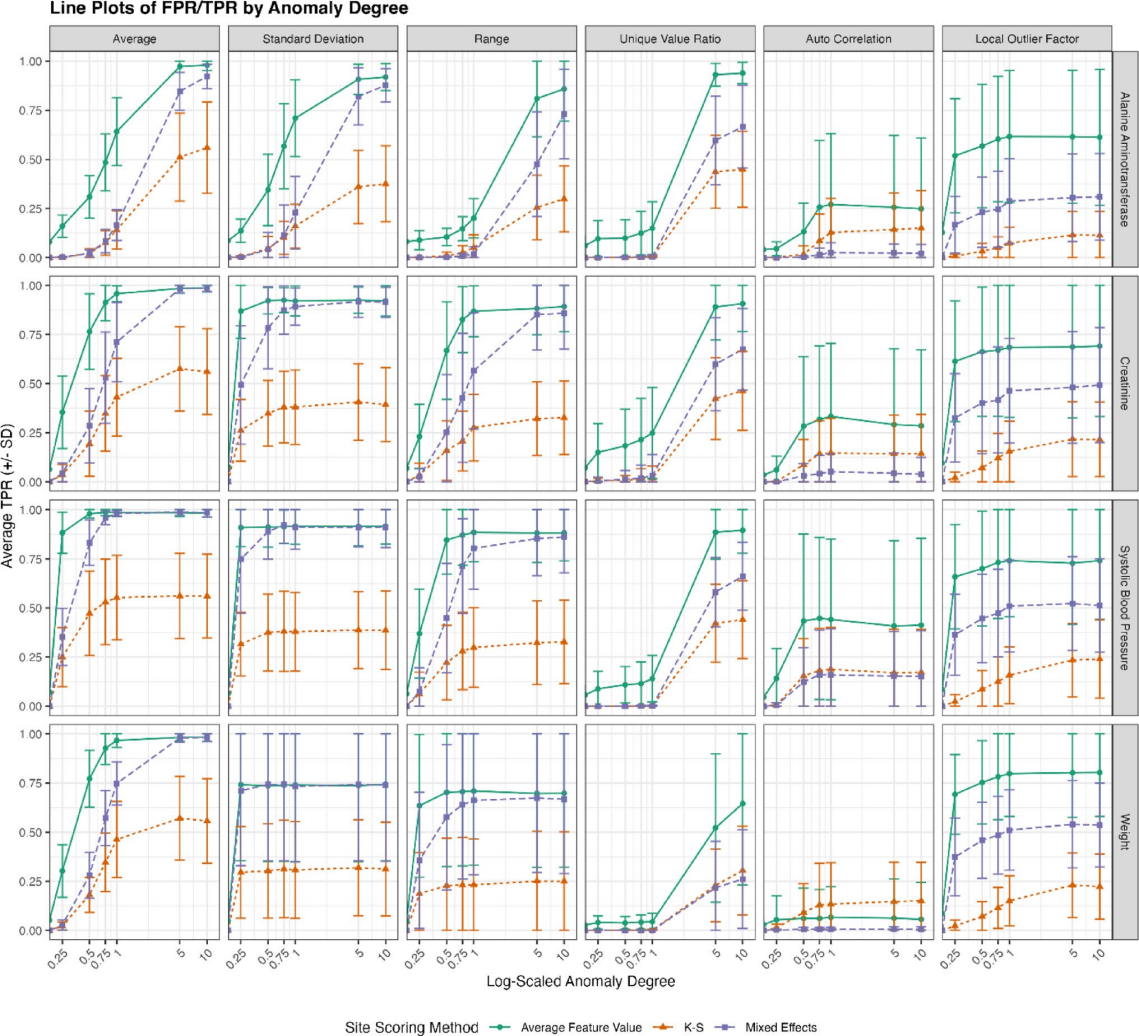
True positive rates (TPR, recall) across different simulation scenarios. Rates are represented as the mean with standard deviation obtained by 15 replicates obtained from simulations based on different studies. Timelines from two laboratory (Alanine Aminotransferase, Creatinine) and 2 vitals measurements (Systolic Blood Pressure, Weight) were selected to be included in the simulation. The simulations randomly introduced 3 anomalous sites with increasing anomaly degrees into each iteration which needed to be detected by the CTAS algorithm. Study-level ratios were based on simulations with 100 iterations

**Fig. 8:**
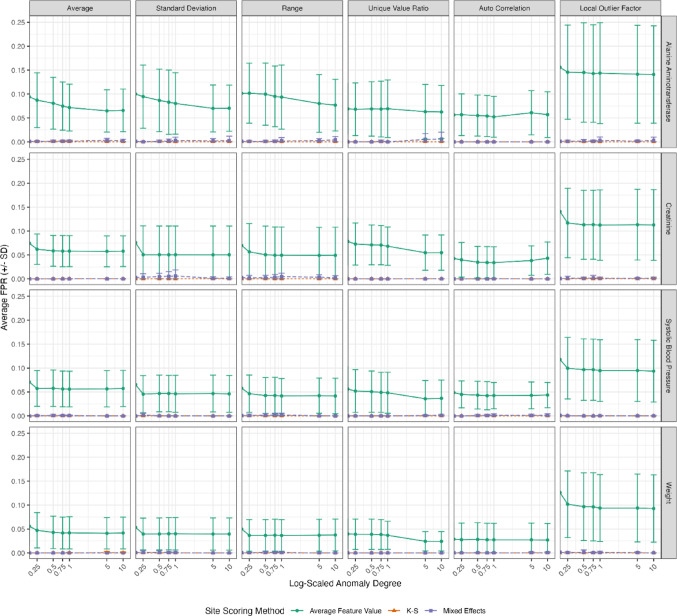
False positive rates (FPR) across different simulation scenarios. Ratios are represented as the mean with standard deviation obtained by 15 replicates obtained from simulations based on different studies. Timelines from two laboratory (Alanine Aminotransferase, Creatinine) and 2 vitals measurements (Systolic Blood Pressure, Weight) were selected to be included in the simulation. The simulations randomly introduced 3 anomalous sites with increasing anomaly degrees into each iteration which needed to be detected by the CTAS algorithm. Study-level ratios were based on simulations with 100 iterations

We tested three different scoring methods to detect six types of site anomalies introduced at varying degrees across 15 study data sets, donated by three different IMPALA members, with 4 measurement types (Alanine Aminotransferase, Creatinine, Systolic Blood Pressure, Weight). The resulting average True Positive Rate (TPR, also known as Recall) and False Positive Rate (FPR), along with their respective standard deviations, were plotted in Figs. 7 and 8. The figure shows that TPR rates generally increase with higher anomaly rates, while the FPR either remains flat or slightly decreases as the degree of anomaly increases.

Overall, the mixed effects modeling method emerged as the most robust approach for detecting site-level anomalies, providing a good balance between high TPR and low FPR. The average feature value method, while effective in identifying true positives, was less reliable due to its high false positive rate.

Among the three site flagging methods, we observed that the average feature value method achieved the highest TPR, detecting almost all high-degree anomalies. However, it also had significantly higher FPR rates compared to the K-S method and mixed effects modeling. The K-S method had the lowest TPR but almost zero FPR. Mixed effects modeling demonstrated the best overall performance, with a TPR surpassing that of the K-S method and a negligible rate of false positives.

We did not observe strong differences between the four different measurement types (Alanine Aminotransferase, Creatinine, Systolic Blood Pressure, Weight). Some were more susceptible to anomalies, resulting in higher TPR at lower anomaly degrees. This is expected, as anomalies were introduced by multiplying existing measurements, which are more sensitive to higher starting values.

Further, we observed an overall decrease in the TPR for weight, specifically for the unique value ratio and autocorrelation anomalies. Weight measurements are unique because three out of 15 studies had only one planned weight measurement per patient. Autocorrelation and unique value count ratio require time series with at least two timepoints to become detectable and cannot be introduced into a time series of length one. On top of that, weight results tend to have a high frequency of rounding, leading to fewer unique values per time series. TPR for weight for the other anomaly time series pairs is not affected by these specific characteristics of weight measurements.

In general, we achieved high TPR rates using mixed effect models except for autocorrelation and LOF where we detected fewer than 50% of all anomalous sites. Suggesting, that these features are more sensitive to artifacts in our study data (e.g. number of patients, time series length, ratio of missing values).

This underscores the importance of considering the nature of the data when interpreting the performance of anomaly detection methods.

## Discussion

Altogether this evaluation of the CTAS algorithm has shown that site-level timeseries anomalies can be detected by comparing summary statistics of individual timeseries from one site against those obtained from all other patients from the study. Out of all site flagging methods we tested, mixed effect modelling has shown the most robust TPR-FPR trade-off.

Mixed effects models can account for region-level and country-level demographic factors when scoring sites. By incorporating higher-level random effects, the model can more accurately detect site-specific biases while controlling for broader demographic influences. This has not been accounted for in our simulations as we have removed all regional and site-specific signals from our test data sets. These abilities might lead to increased statistical performance of mixed effect models in practice.

Previously published central SDM strategies select a number of data-type-appropriate statistical tests to identify site anomalies [11–13]. They also employ mixed effect models to account for different variability among sites and multiplicity correction [13, 14] and demonstrate that their application can improve data quality over time [3]. Presently there is no open-source application for these methods.

In general, small samples tend to have a higher variability than large samples. Thus, small or late starting sites with a low number of subjects or time points are more likely to have extreme values when continuously monitoring ongoing trial data. Most statistical tests will control for a difference in sample size and will reliably flag site outliers among sites with different number of subjects. But smaller differences remain easier to detect in larger samples and differences in smaller samples need to be larger to get detected.

The ratio of small sites with less than 3 patients in an average study can be quite high and has been roughly 20% in the investigated studies. The overall maximum performance depends on how well these sites can be detected therefore we chose not to exclude any sites although others have excluded sites with only one patient [13]. This is why we see a lower maximum TPR when using the K-S site scoring method because it uses a rank-based test that is somewhat indifferent to the magnitude of the outlier. The mixed effect model scoring method, also employed by others [13, 14], accounts for sample number and magnitude but assumes a normal distribution and has an increased FPR compared to the non-parametric K-S method. To account for the lower TPR that can be expected for small sites we recommend to also check for patient-level outliers using similarity plot as depicted in Fig. 4.

Sample-size induced variability can also be introduced by differences in the number of subject visits. Some measurements will also depend on procedures planned at different visits determined by the study protocol thus different values can be expected for early screening visits compared to late follow up visits. CTAS controls for this by only comparing summary metrics from time series composed of the same time points. Thus, CTAS avoids errors introduced by comparing aggregates from samples of different sizes and variability introduced by the study protocol.

We have picked four different measurement types (Alanine Aminotransferase, Creatinine, Systolic Blood Pressure, Weight) across 15 studies to investigate the variability of the results. We did observe slightly different TPR rates across the different anomalies that were introduced.

Weight stood out as the TPR was reduced for unique value ratio and autocorrelation. We attribute this to a prevalence of rounding and that three out of 15 studies only had one planned weight measurement per patient. For unique value ratio, the reduced TPR is therefore caused by several “control” sites with heterogenous round practices making it difficult to distinguish them from sites with injected artificial anomalies. Nevertheless, other anomalies could be efficiently detected. These observations lead to the conclusion that, dependent on the homogeneity and precision of measurements, CTAS can either detect sites lacking precision or, if the precision is homogenous, it can detect sites with duplicates.

The artificial anomalies that we have introduced (Fig. 5) are extreme and should significantly alter the time series features used by CTAS to detect outliers. However, autocorrelation feature requires longer time series and is sensitive to missing values. Similarly, for LOF there will be sensitive to the total number of subjects per site, their time series length and their ratio of missing values. This shows in the results, where we do not manage to achieve high detectability rates for these anomalies. We cannot fully characterize all possible edge cases and have thus combined data from 15 studies to demonstrate the average TPR we can expect in a clinical trial scenario.

In practice extreme values in the data set will not only change one but many time series features. Thus, there is a good chance that anomalies in the data will create multiple signals that complement each other. CTAS does not try to preemptively select the most appropriate statistical test for a given measurement type and time series length. Therefore, all timeseries features can always be measured across all timeseries to cast a wide net for detecting anomalies.

CTAS can only detect anomalies that impact the time series features (average, SD, range, unique value count, autocorrelation and LOF) real-world examples can be found in Table 1. Therefore, we only tested CTAS against artificial anomalies we believe to mimic real-world anomalies. However, we are lacking a data set with labelled real-world examples and thus resort to detecting artificial anomalies within a data set with realistic data patterns. CTAS does not offer built-in native support for categorical, ordinal and date type data they can be transformed to a numerical data type before running CTAS. This is expected to provide similar results as specifically adapted tests for these data types although this remains to be tested but would require a different strategy for introducing anomalies which would exceed the scope of this work.

In practice, many site anomalies identified by CTAS are often linked to data collection differences allowed by the study protocol or variations in patient populations. Reviewing the anomalies therefore requires knowledge of the protocol and awareness of existing data quality control strategies. A risk-based review of the anomalies based on data criticality is appropriate to limit false positive fatigue. We recommend a CTAS score threshold of 1.3 (*p*-value: ~ 0.05) to perform a due-diligence review for detecting unexpected anomalies. TPR and FPR rates presented in this work are only applicable to the experimental conditions. They serve as a benchmark for the rates obtained in practice where CTAS scores might be aggregated differently. Threshold selection could further be refined by employing a loss function accounting for the cost and benefit of the TPR and FPR which requires prior knowledge about the nature of the anomalies for each measurement. For expected anomalies, we recommend more traditional data quality control methods such as edit checks.

## Conclusion

Current regulatory guidelines promote the use of proportionate RBQM in clinical trials to support traditional onsite monitoring, medical data review and data management activities. SDM can be used to detect unexpected anomalies within clinical trial data as part of the RBQM activities.

We have demonstrated that the CTAS algorithm can be used to detect site-level anomalies of various kinds and that its accuracy increases the more extreme the anomalies become. Among all site flagging methods tested, the mixed effect model option has been shown to possess the best TPR to FPR ratio. We also demonstrated that CTAS can be used across different types of clinical time series measurements, but we discuss limitations for time series that are short, possess higher ratios of missing values or are not always recorded with the same precision. Within an RBQM framework, CTAS can screen clinical data for site anomalies and flag cases for manual review. The higher the score the more likely a site is to represent a genuine outlier that can be confirmed in a time series data visualization. The algorithm can be adapted to all data types covering the entire clinical data set, blind spots not covered by manual risk mitigation strategies can be avoided, possible unforeseen scenarios missed by first line control activities (programmed edit checks) can be detected.

Similar tools have been shown to improve data quality over time [3]. CTAS having the advantage of being better adapted to time series data. The CTAS R package is also available as an open-source R package and provides greater transparency than individual proprietary solutions. By promoting the adoption of CTAS among IMPALA’s 19 industry members and enhancing compatibility with other open-source RBQM tools [4], we aim to contribute to the advancement of robust, transparent site anomaly detection in clinical trials.

## Supplementary Information

The CTAS R package is available on github [16] as version v0.4.0. The code written for the validation is available in a separate repository [17] version v0.0.7.

## Data Availability

The data supporting the findings of this study are available from the authors and may be shared upon reasonable request placed via the corresponding author. As the data are owned by multiple institutions, responses to data-sharing requests may vary depending on the policies of each institution.

## References

[1] ICH E6 Good clinical practice - Scientific guideline | European Medicines Agency (EMA) 2002. https://www.ema.europa.eu/en/ich-e6-good-clinical-practice-scientific-guideline. Accessed 23 Jan 2026.

[2] Schmidt CO, Struckmann S, Enzenbach C, Reineke A, Stausberg J, Damerow S, et al. Facilitating harmonized data quality assessments. A data quality framework for observational health research data collections with software implementations in R. BMC Med Res Methodol. 2021;21:63. 10.1186/s12874-021-01252-7. 33810787 10.1186/s12874-021-01252-7 PMC8019177

[3] de Viron S, Trotta L, Steijn W, Young S, Buyse M. Does central statistical monitoring improve data quality? An analysis of 1,111 sites in 159 clinical trials. Ther Innov Regul Sci. 2024;58:483–94. 10.1007/s43441-024-00613-w. 38334868 10.1007/s43441-024-00613-w PMC11043176

[4] Wu G, Childress S, Wang Z, Roumaya M, Stern CM, Dickens C, et al. Good statistical monitoring: a flexible open-source tool to detect risks in clinical trials. Ther Innov Regul Sci. 2024;58:838–44. 10.1007/s43441-024-00651-4. 38722529 10.1007/s43441-024-00651-4 PMC11335794

[5] Ménard T, Young K, Siegel L, Emerson J, Studt R, Sidor L. Cross-company collaboration to leverage analytics for clinical quality and accelerate drug development: the IMPALA industry group. CPT Pharmacomet Syst Pharmacol. 2021;10:799–803. 10.1002/psp4.12677. 10.1002/psp4.12677 PMC8376133 34247450

[6] Breunig MM, Kriegel H-P, Ng RT, Sander J. LOF: identifying density-based local outliers. SIGMOD Rec. 2000;29:93–104. 10.1145/335191.335388.

[7] Benjamini Y, Yekutieli D. The control of the false discovery rate in multiple testing under dependency. Ann Stat. 2001;29:1165–88. 10.1214/aos/1013699998.

[8] Schröer G, Trenkler D. Exact and randomization distributions of Kolmogorov-Smirnov tests two or three samples. Comput Stat Data Anal. 1995;20:185–202. 10.1016/0167-9473(94)00040-P.

[9] Vinutha HP, Poornima B, Sagar BM. Detection of outliers using interquartile range technique from intrusion dataset. In: Satapathy SC, Tavares JMRS, Bhateja V, Mohanty JR, editors. Information and decision sciences. Singapore: Springer; 2018. p. 511–8. 10.1007/978-981-10-7563-6_53.

[10] Mancini E, G G, Zhang K, Kumari P, Bundfuss S, Zhu Z, et al. pharmaversesdtm: SDTM Test Data for the “Pharmaverse” Family of Packages 2025.

[11] Venet D, Doffagne E, Burzykowski T, Beckers F, Tellier Y, Genevois-Marlin E, et al. A statistical approach to central monitoring of data quality in clinical trials. Clin Trials. 2012;9:705–13. 10.1177/1740774512447898. 22684241 10.1177/1740774512447898

[12] Kirkwood AA, Cox T, Hackshaw A. Application of methods for central statistical monitoring in clinical trials. Clin Trials. 2013;10:783–806. 10.1177/1740774513494504. 24130202 10.1177/1740774513494504

[13] Trotta L, Kabeya Y, Buyse M, Doffagne E, Venet D, Desmet L, et al. Detection of atypical data in multicenter clinical trials using unsupervised statistical monitoring. Clin Trials. 2019;16:512–22. 10.1177/1740774519862564. 31331195 10.1177/1740774519862564

[14] Desmet L, Venet D, Doffagne E, Timmermans C, Burzykowski T, Legrand C, et al. Linear mixed-effects models for central statistical monitoring of multicenter clinical trials. Stat Med. 2014;33:5265–79. 10.1002/sim.6294. 25213096 10.1002/sim.6294

[15] IMPALA Consortium | Inter-Company Quality Analytics. IMPALA Consortium n.d. https://impala-consortium.org/. Accessed 23 Jan 2026.

[16] IMPALA-Consortium/ctas: Time Series Outliers and Anomalies n.d. https://github.com/IMPALA-Consortium/ctas. Accessed 23 Jan 2026.

[17] IMPALA-Consortium/ctasval n.d. https://github.com/IMPALA-Consortium/ctasval. Accessed 23 Jan 2026.

